# Hypertrophic cardiomyopathy: Sudden cardiac death risk stratification in adults

**DOI:** 10.21542/gcsp.2018.25

**Published:** 2018-08-12

**Authors:** Paloma Jordà, Ana García-Álvarez

**Affiliations:** Cardiology Department, Institut Clínic Cardiovascular, Hospital Clínic, Universitat de Barcelona, Institut d’Investigacions Biomèdiques August Pi i Sunyer (IDIBAPS), Barcelona, Spain

## Introduction

Sudden cardiac death (SCD) is a devastating and often unpredictable complication of hypertrophic cardiomyopathy (HCM) that may occur as the initial disease presentation, frequently in asymptomatic or mildly symptomatic young people. Until 2000, only small series of patients examining predictors of SCD had been published, with a selection bias towards severe disease. Subsequently, larger series that are more representative of the HCM spectrum have shown that the annual SCD rate is less than 1%^1,2^, and that there are subgroups of patients with a clearly higher risk.

**Table 1:** Overview of studies and their main findings discussed in this review.

Articles	Definition of the risk factor	N	Follow-up (years) Mean ± SD or median (range)	Incidence of SCD	Univariate HR (95%CI) or *p* value	Multivariate HR (95%CI) or *p* value
**Age** (1 study demonstrated statistically significant independent association*)						
Maki et al.^31^	Continuous variable (years)	309	9.4 (2–25)	28/309	0.99 (0.97–1.02)	No data
Sorajja et al.^44^	Continuous variable (years)	433	5.9 (0.1–25.3)	29/433	No data	1.01 (0.98–1.03)
Kofflard et al.^17^	Continuous variable (years)	225	7.5 ± 7	20/225	No data	NS (numerical data NA)
Maron MS et al.^45^	<20, 20-39, 40-60, >60 years	1101	6.3 ± 6.2	71/1101	No data	NS (numerical data NA)
*Spirito et al.^19^	<18, 18-39, ≥40 years	1511	5.6 ± 5.2	74/1511	No data	0.63 (0.32–1.22) and 0.29 (0.15–0.57) for 18–39 and ≥40 categories, respectively
**Sex** (No study demonstrated statistically significant independent association)						
Maki et al.^31^	No definition	309	9.4 (2–25)	28/309	0.88 (0.10–1.65)	No data
Sorajja et al.^44^	Female gender	433	5.9 (0.1–25.3)	29/433	No data	1.48 (0.68–3.21)
Maron MS et al.^45^	Female gender	1101	6.3 ± 6.2	71/1101	No data	*p* = 0.75, other data NA
Olivotto et al.^36^	Female gender	969	6.2 ± 6.1	59/969	No data	*p* = 0.97, other data NA
**Non-sustained ventricular tachycardia (NSVT) on Holter** (3 studies demonstrated statistically significant independent association*)						
Maki et al.^31^	HR ≥120 bpm lasting <30 seconds within 24 h	309	9.4 (2–25)	28/309	1.61 (0.47–2.76)	NS, numerical data NA
Kofflard et al.^17^	Sustained or NSVT within 24 h	225	7.5 ± 7	20/225	No data	NS, numerical data NA
Elliott et al.^24^	≥ 3 beats at HR ≥120 bpm <30 seconds within 48 h	368	3.6 ± 2.5	22/368	1.8 (0.7–4.7)	1.9 (0.7–5.0)
*Montserrat et al.^20^	≥3 beats at HR ≥ 120 bpm <30 seconds within 24 h to 48 h	532	5.8 ± 3.3	32/532	No data	2.8 (1.4–5.6)
*Elliott et al.^32^	≥3 beats at HR ≥ 120 bpm <30 seconds within 48 h	917	5 (2.5–8.3)	54/917	No data	3.84 (2.1–7.0)
*Gimeno et al.^21^	≥3 beats at HR ≥ 120 bpm <30 seconds within 24 h to 48 h	1380	4.5 ± 4	No data	No data	2.57 (1.55–4.26)
**Severe hypertrophy on echocardiography** (4 studies demonstrated statistically significant independent association*)						
Maki et al.^31^	Continuous variable (septal thickness in mm)	309	9.4 (2–25)	28/309	1.03 (0.97–1.09)	No data
Kofflard et al.^17^	≥25 mm	225	7.5 ± 7	20/225	No data	NS (numerical data NA)
Maron MS et al.^45^	≥30 mm	1101	6.3 ± 6.2	71/1101	No data	*p* = 0.82 (other data NA)
*Spirito et al.^19^	≤10, 11-15, 16-19, 20-24, 25-29, 30-34, ≥35 mm	1511	5.6 ± 5.2	74/1511	No data	*p* = 0.04 for strata comparison
*Elliott et al.^24^	≥ 30mm	368	3.6 ± 2.5	36/368	4.1 (1.7–9.5)	2.9 (1.1–7.1)
*Montserrat et al.^20^	≥ 30 mm in patients ≤30 years old	532	5.8 ± 3.3	32/532	No data	3.5 (1.2–10.7)
Elliott et al.^32^	≥ 30 mm	917	5 (2.5–8.3)	54/917	No data	1.70 (0.8–3.8)
Gimeno et al.^21^	≥ 30 mm	1380	4.5 ± 4	No data	No data	0.90 (0.42–1.93)
*Spirito et al.^25^	≤10, 11-15, 16-19, 20-24, 25-29, 30-34, ≥35 mm	480	6.5 ± 4.6	23	*p* = 0.001	1.76 (1.19–2.60)
**Abnormal blood pressure response to exercise** (1 study demonstrated statistically significant independent association; different definition**)**						
* Maki et al.^31^	Exercise-induced change in SBP	309	9.4 (2–25)	28/309	0.97 (0.96–0.99)	*p* < 0.05 (other data NA)
Elliott et al.^24^	Failure to ↑ SBP 25 mmHg from baseline to peak exercise or ↓ 15 mmHg during exercise & ≤40 years old	368	3.6 ± 2.5	36/368	2.4 (1.0–5.5)	1.8 (0.7 to 4.4)
Montserrat et al.^20^	SBP failed to ↑ >25 mmHg from baseline, or ↓ >10 mmHg from maximum BP during exercise & ≤30 years old	532	5.8 ± 3.3	32/532	No data	0.5 (0.2–1.7)
Elliott et al.^32^	SBP failed to ↑ >25 mmHg from baseline, or ↓ >10 mmHg from maximum BP during exercise & ≤40 years old	917	5 (2.5–8.3)	54/917	No data	1.42 (0.7–2.8)
Gimeno et al.^21^	SBP failed to ↑ >25 mmHg from baseline, or when ↓ >10 mmHg from maximum BP during exercise & ≤40 years old	1380	4.5 ± 4	No data	No data	1.43 (0.86–2.36)
**Family history of SCD (FH-SCD)** (2 studies demonstrated statistically significant independent association*)						
Maki et al.^31^	FH-SCD judged to be probably due to HCM	309	9.4 (2–25)	28/309	2.44 (1.62–3.26)	NS (numerical data NA)
Kofflard et al.^17^	FH-SCD in a first degree relative at age <40 years old	225	7.5 ± 7	20/225	No data	NS (numerical data NA)
Elliott et al.^24^	FH-SCD in ≥ 2 first-degree relatives <40 years old	368	3.6 ± 2.5	36/368	1.9 (0.8–4.5)	5.3 (1.9–14.9) for FHSD *and* syncope
Montserrat et al.^20^	FH-SCD in ≥ 2 first-degree relatives in patients ≤ 30 years old	532	5.8 ± 3.3	32/531	No data	1.4 (0.5–4.5)
*Elliott et al.^32^	FH-SCD in relatives <40 years old	917	5 (2.5–8.3)	54/917	No data	1.88 (1.0–3.5)
*Gimeno et al.^21^	FH-SCD in relatives <40 years old	1380	4.5 ± 4	No data	No data	1.79 (1.09–2.94)
Spirito et al.^25^	FH-SCD ≥1 member with HCM or if one or more close relatives without documented HCM died suddenly at <50 years of age	480	6.5 ± 4.6	23	2.1 (0.0–11.9)	No data
**Unexplained syncope** (4 studies demonstrated statistically significant independent association*)						
Maki et al.^31^	No definition	309	9.4 (2–25)	28/309	2.20 (1.30–3.11)	NS, numerical data NA
*Kofflard et al.^17^	No definition	225	7.5 ± 7	20/225	No data	4.3 (1.8–5.9)
*Spirito et al.^19^	Syncope within previous 6 months	1511	5.6 ± 5.2	74/1511	No data	4.89 (2.19–10.94)
Elliott et al.^24^	≥ 1 episodes of syncope within 12 previous months	368	3.6 ± 2.5	36/368	2.0 (0.8 to 4.9)	5.3 (1.9 to 14.9) for FH-SCD *and* syncope
Montserrat et al.^20^	Syncope within previous 12 months in individuals ≤ 30 years old	532	5.8 ± 3.3	32/531	No data	1.3 (0.4–4.3)
*Elliott et al.^32^	Recurrent unexplained syncope within previous 12 months	917	5 (2.5–8.3)	54/917	No data	2.27 (1.2–4.2)
*Gimeno et al.^21^	No definition	1380	4.5 ± 4	No data	No data	2.08 (1.21–3.56)
**Left ventricular outflow tract obstruction** (Measured by Echo-Doppler, except in one study. 5 studies demonstrated statistically significant independent association*)						
*Maki et al.^31^	Invasive measurement ≥ 30 mmHg at rest	309	9.4 (2–25)	28/309	1.01 (1.01–1.02)	*p* < 0.05 (numerical data NA)
Kofflard et al.^17^	≥50 mmHg at basal conditions	225	7.5 ± 7	20/225	No data	NS (numerical data NA)
*Maron MS et al.^45^	≥ 30 mmHg at rest	1101	6.3 ± 6.2	71/1101	2.1 (1.1–3.7)	1.9 (1.1–3.5)
*Olivotto et al.^36^	≥30 mmHg at rest	969	6.2 ± 6.1	59/969	No data	1.84 (1.14–2.98)
*Elliott et al.^32^	≥90 mmHg	917	5 (2.5–8.3)	54/917	No data	3.82 (1.6–9.2)
*Gimeno et al.^21^	≥90 mmHg	1380	4.5 ± 4	No data	No data	2.41 (1.08–5.53)
Spirito et al.^25^	≥30 mmHg at basal conditions	480	6.5 ± 4.6	23	NS, *p* = 0.63 (comparison of incidences)	NS, *p* = 0.76 (comparison of incidences)
**Atrial fibrillation (AF)** (1 study demonstrated statistically significant independent association*)						
Maki et al.^31^	No definition	309	9.4 (2–25)	28/309	0.4997 (−0.939–1.938)	No data
*Sorajja et al.^44^	Chronic AF	433	5.9 (0.1–25.3)	29/433	No data	4.90 (1.49–16.67)
Kofflard et al.^17^	Persistent AF	225	7.5 ± 7	20/225	No data	NS (numerical data NA)
Maron MS et al.^45^	Paroxysmal or chronic AF	1101	6.3 ± 6.2	71/1101	No data	NS, *p* = 0.72 (other data NA)
**Left atrial size on echocardiography** (1 study demonstrated statistically significant independent association*)						
Maki et al.^31^	Continuous variable (mm)	309	9.4 (2–25)	28/309	1.06 (1.00–1.12)	No data
Spirito et al.^25^	<40 mm, 40–50 mm, >50 mm	480	6.5 ± 4.6	23/480	NS, *p* = 0.30 (comparison of incidences)	NS, *p* = 0.21 (other data NA)
*Spirito et al.^19^	Continuous variable (mm)	1511	5.6 ± 5.2	74/1511	No data	1.03 (1.00–1.06)

**Notes.**

Abbreviations SD Standard deviation NS Non-significant NA Not available HR Heart rate SBP Systolic blood pressure BPM Beats per minute

## Pathophysiology of SCD

Genetic and molecular substrate, myofibrillar disarray, ventricular hypertrophy, microvascular ischemia and fibrosis^3^ predispose patients with HCM to re-entrant ventricular arrhythmias^4^. Disruption of intercalated discs, increased myofilament calcium sensitivity and abnormal calcium handling are additional arrhythmogenic factors^5,6^. Life-threatening ventricular tachyarrhythmias can be triggered by a number of environmental factors (e.g., intense physical exertion) or features intrinsic to the disease process, including left ventricular outflow obstruction, systemic arterial hypotension and supraventricular tachyarrhythmias. Systematic analysis of stored electrograms has shown that most ventricular arrhythmias occur spontaneous in normal sinus rhythm, sometimes precipitated by premature ventricular complexes^7^, although rapid atrial fibrillation has also been demonstrated as a trigger^8,9^.

## Risk stratification and prevention of SCD

Implantable cardioverter defibrillators (ICD) are effective in terminating life-threatening ventricular arrhythmias in HCM, whereas pharmacologic therapy has not been demonstrated to provide protection from SCD. ICD implantation in secondary prevention is rarely a clinical challenge^10^, but identifying patients within this heterogeneous disorder who are at high risk of SCD when prior to a first event is a challenge. Clinical guidelines recommend that all patients should undergo SCD risk stratification at their initial evaluation and periodically thereafter. The clinical parameters that associate with SCD are summarized in Table 1 and discussed below.

## Risk factors and modifiers

### 1. Prior personal history of aborted ventricular fibrillation or sustained ventricular tachycardia (VT)

Patients with HCM who have experienced sustained ventricular arrhythmia represent the highest risk for subsequent arrhythmic events, with an approximate recurrence rate of 10% per year, although it has been shown that some patients may have no repeated events or have decades-long arrhythmia free intervals between episodes^11,12^.

### 2. Family history of SCD

A family history of SCD is associated with an increased risk of SCD in other affected family members, particularly if there are multiple SCD events and if they occurred in young people^13,14^. Nonetheless, discrepancy exists regarding the independent relationship between family history of SCD and risk for the individual patient^15^, probably related to the relative low incidence of events and the variability in the definition of family history of SCD. The average Hazard ratio (HR) from a systematic review was 1.27, 95% confidence interval (CI) 1.16–1.38.

### 3. Unexplained syncope

Syncope, defined as a temporary loss of consciousness secondary to transient global cerebral hypoperfusion, is a challenging clinical diagnosis. There are many potential causes of syncope in HCM including sustained ventricular arrhythmias, supraventricular tachycardias, bradyarrhythmias and exercise-related left ventricular outflow tract obstruction (LVOTO). Patients may also experience vasovagal syncope. The difficulty in determining the cause of a temporary loss of consciousness means that syncope alone has a low sensitivity and specificity for SCD. In a systematic review, the average HR of unexplained syncope was 2.68 but the association did not reach statistical significance (95% CI [0.97–4.38])^16^. Therefore, a careful clinical history is required before it can be considered a potential marker for SCD. It is particularly important when it is exertional or recurrent, it occurs in the young, or in the recent past (<6 months)^17–19^.

### 4. Non-sustained ventricular tachycardia (NSVT)

NSVT, defined as >3 beats at >120 bpm, is a common phenomenon in HCM. The HR for the association between SCD and NSVT from a systematic review was 2.89 (95% CI [2.2–3.6])^16^. However, different inclusion criteria have been applied in published studies, in others a large number of patients had no ECG ambulatory recordings at all. Limited data suggest that the association with SCD is strongest in individuals under 30 years of age. The OR for SCD at 5 years in patients ≤ 30 with NSVT was 4.35 (95% CI [1.54–12.28]), as compared to 2.16 (96% CI [0.82–5.69]) in those older than 30 years of age^20^.

Exercise-induced ventricular arrhythmias, present in 1-2% of patients with HCM, have also been reported to be independently associated with SCD (adjusted HR=3.14; 95% CI [1.29–7.61])^21^.

### 5. Maximum left ventricular wall thickness

The magnitude of left ventricular hypertrophy correlates with the risk of SCD^22,23^. This is to be expected given the potential impact of that thickening on myocardial replacement scarring resulting from intramural small vessel disease and mass-to-coronary flow mismatch, creating an electrophysiologically unstable substrate.

Several large studies have shown an independent association between a magnitude of hypertrophy ≥30 mm and SCD^24,25^ including a systematic review (HR=3.10, 95% CI [1.81–4.40])^16^. However, this cut-off value is somehow arbitrary and not based on any specific biological properties and the risk estimate does not abruptly increase for patients with ≥30 mm wall thickness but rather increases in a linear^25^ or quadratic fashion^26^ and appears to carry more prognostic value in younger patients.

### 6. Abnormal blood pressure response during exercise

An inappropriate systemic systolic blood pressure (BP) response during exercise testing, defined as either a failure to increase by at least 20 mmHg or a drop of at least 20 mmHg during effort, is common in patients with HCM^27,28^ and seems to be related with LVOTO. It is suggested^29,30^ that an inappropriate drop in systemic vascular resistance, despite an appropriate increase in cardiac output is a contributory mechanism. Two studies showed a univariate association between this abnormal response and subsequent SCD^28,31^. However, no study using this definition has demonstrated a significant independent prognostic value. In a systematic review, the association was not statistically significant (HR 1.3, 95% CI [0.64–1.96])^16^.

### 7. Left ventricular outflow tract obstruction (LVOTO)

There is evidence for a higher risk of SCD among patients with left ventricular outflow tract gradients ≥30 mmHg^31,32^ and a positive correlation between the magnitude of risk and the severity of obstruction^32^. LVOTO can provoke SCD either by causing severe reduction in cardiac output leading to electromechanical dissociation or by precipitating ventricular arrhythmias though myocardial ischemia caused by increased left ventricular end-diastolic pressure^33^. Conversely, relief of LVOTO through surgical myectomy is associated with low subsequent rates of SCD^34,35^.

Due to its dynamic nature, LVOTO was not originally considered in risk scores. The fact that it can be strongly mitigated by drugs or septal reduction also has the potential to dilute its contribution to SCD.

### 8. Age and gender

While there appears to be no difference in SCD rates based on gender, age represents an important factor. SCD is more common in younger patients, especially those under the age of 35 year; however, up to 20% of SCDs occur in patients over the age of 65^36^. As discussed NSVT^30^ and left ventricular hypertrophy (LVH) appear more significant as risk factors in younger patients^25^.

### 9. Late gadolinium enhancement on cardiac magnetic resonance imaging

Late gadolinium enhancement on cardiac magnetic resonance (LGE-CMR) is used as a surrogate for the degree and distribution of fibrosis, but its positive predictive value for ventricular arrhythmias and SCD in HCM patients is controversial.

LGE-CMR is present in up to 70% of cases, and is associated with impaired systolic function and other risk factors for SCD, in particular NSVT^37,38^. Two recent meta-analysis that assessed prognostic value of LGE-CMR in HCM concluded that it was a predictor of SCD irrespective of LV ejection fraction^39,40^. However, due to its high prevalence, binary analysis of myocardial fibrosis does not aid in decision-making. LGE-CMR extension is also influenced by the imaging protocol used, and therefore, there is no consensus for considering LGE-CMR an independent predictor for SCD.

### 10. Left ventricular apical aneurysm

Approximately 2–5% of patients with HCM, typically those with mid-ventricular hypertrophy, develop a thin-walled left ventricular apical aneurysm associated with regional scarring. A higher incidence of clinical events during follow-up have been reported in this subgroup, including a documented risk of SCD of 5% per year^41,42^.

### 11. Genetic mutations

Early studies of HCM pedigrees suggested that some mutations in cardiac beta-myosin heavy chain and in troponin-T were associated with a higher incidence of premature death, decreased life expectancy, and early onset disease manifestations than others^43^. However, subsequent studies from unselected consecutive patients did not confirm a clear prognostic association, although most were underpowered to investigate the association between individual mutations and risk. Future advances in genotype-based risk stratification will probably shed light in the management of these patients.

### 12. Supraventricular arrhythmias and left atrial size

Atrial fibrillation (AF) and left atrial size reflecting LV filling pressures and remodeling may indirectly reflect disease progression and SCD risk. Left atrial diameter has been associated with SCD in a single study with a HR 1.03; 95% CI [1.00–1.05], *p* = 0.04^19^. Chronic – but not paroxysmal or persistent – AF has also been associated with SCD^17,31,44,45^.

In a study of 71 HCM individuals with ICD for primary or secondary prevention, ventricular fibrillation or rapid VT episodes (>200 bpm) were more frequently preceded by supraventricular rhythms greater than 100 bpm (*p* = 0.001) suggesting that supraventricular tachycardia might play a role in the trigger of rapid VT^46^.

### 13. Other factors

Electrophysiologic testing with programmed ventricular stimulation has not demonstrated utility in identifying those HCM patients at higher risk for SCD because the induction of VT or ventricular fibrillation is highly dependent on the aggressiveness of the stimulation protocol. Therefore, electrophysiologic testing with programmed ventricular stimulation is not recommended on a routine basis but may be useful in selected patients with unexplained syncope.

## Guidelines recommendations for ICD implantation: 2003 to 2018

The ACC/ESC Expert Consensus published in 2003^47^ recommended ICD in individuals with two or more major risk factors but stated that “strong consideration should be afforded for a prophylactic ICD” if a single major risk factor was present. The following major risk factors were considered: prior cardiac arrest or spontaneously occurring sustained VT; family history of a premature HCM-related SCD; unexplained syncope, particularly in young patients or when exertional or recurrent; extreme LV hypertrophy with a maximum wall thickness of 30 mm or more, particularly in adolescents and young adults; abnormal BP response during upright exercise (greater predictive value in patients less than 50 years old or if hypotensive); and NSVT on ambulatory ECG recordings. Minor risk factors were the identification of a high-risk mutant gene, LVOTO, atrial fibrillation, myocardial ischemia and intensive (competitive) physical exertion.

In an update of these guidelines, The ACC/AHA/ESC 2006 guidelines for management of patients with ventricular arrhythmias^48^ and the ACC/AHA/HRS 2008 guidelines for device-based therapy of cardiac rhythm abnormalities^49^ stated that ICD implantation could be effective or reasonable for patients with HCM who had one or more major risk factors for SCD (both class IIa recommendations, level of evidence C). The 2011 ACCF/AHA guidelines^50^ also recommended implantation of an ICD if a single risk factor such as family history of SCD, LV thickness greater than 30 mm or recent unexplained syncope, was present whereas the relevance of NSVT and abnormal BP response was downgraded, and required the concomitant presence of additional risk factors such as left ventricular outflow obstruction, LGE-CMR, apical aneurysm or double mutations to recommend an ICD.

There is agreement that there is a correlation between the sum of risk factors and the incidence of SCD, and that individual risk factors in insolation have poor positive predictive value for SCD. However, validation studies have shown that the simple summation model has a relatively poor predictive performance and leads to the implantation of ICD in patients at low risk^51^. Moreover, previous algorithms have ignored the influence of age and consider variables such as myocardial thickness and LVOTO as binary factors when they are associated with a continuum of risk.

In 2014, the European Society of Cardiology^52^ published a new score based on a multicenter, retrospective cohort study that included 3.675 individuals^26^ in which risk factors independently associated with SCD in at least one multivariate study analysis were evaluated. Statistical modelling was employed to find clinical variables that were associated with SCD at ≥0.15 significance, resulting in exclusion of LV ejection fraction and abnormal BP response but including LVOTO, age and size of the left atrium. The final model was used to generate an online calculator [http://www.doc2do.com/hcm/webHCM.html] designed to provide individualized 5 year risk estimates for SCD. In this score, age, myocardial thickness, left atrial size, and LVOTO are treated as continuous variables. The 2014 ESC Guidelines use the model to generate consensus based recommendations for ICD implantation.

Concerns about the sensitivity of the ESC SCD calculator as compared with the 2011 ACCF/AHA guidelines were raised in a study of 1.629 patients previously risk-stratified according to the ACCF/AHA guidelines. The authors found that the ESC calculator had adequate specificity but poor sensitivity compared to the ACCF/AHA guidelines^55^. However, in a global validation study published in 2018^53^, SCD was observed in 1.4% of individuals in the low risk group (estimated risk of <4%) and in 8.9% of individuals in the high risk group (estimated 5 year risk ≥6%). The number needed to treat (NNT) of ICD required to save one live was 13. The score has shown a markedly higher calibration and discrimination than previous algorithms^54^.

In the latest ESC clinical practice guidelines, LGE-CMR was included as an additional parameter that can be considered in decision-making among patients with intermediate risk score along with the presence of apical aneurysm, LV ejection fraction <50%, double mutations or NSTV during exertion.

The HCM-SCD score is not validated in children or in individuals with myocardial hypertrophy due to metabolic diseases or syndromes with multi-organ involvement. Individuals with LV hypertrophy higher than 35 mm had a low representation in the population of the score, and therefore, the model should be used with caution in this subgroup of patients. The score has not been validated in patients undergoing a septal reduction therapy, and therefore it is not clear whether the prior estimated risk or the recalculated one after therapy should be used. The defined cut-off point for recommending ICD implantation (estimated risk of sudden death >4–6%) is an arbitrary value that has been assumed by balancing medical and socioeconomical criteria.

## Final considerations

The decision to recommend ICD implantation is complex. It should be based on individual judgement for the particular patient, by taking into account the overall clinical profile including age, the strength of the risks factors identified, the level of risk acceptable to the patient and family, anxiety, and the potential complications related to the lead systems and to inappropriate device discharges. It is relevant to consider the patient’s age, particularly because device complications are more likely in children and young adults over the long period of follow-up^12,56^. Prior to ICD implantation patients should be advised on the risk of inappropriate shocks, implant complications, and the social and occupational implications of an ICD.
